# Early multimodal vasopressor strategy in septic shock (TRICYCLE)—Study protocol for a randomized controlled clinical trial

**DOI:** 10.1371/journal.pone.0331304

**Published:** 2025-08-29

**Authors:** Žiga Kalamar, Mario Gorenjak, Giovanni Landoni, Andrej Markota

**Affiliations:** 1 Medical Intensive Care Unit, University Medical Centre Maribor, Maribor, Slovenia; 2 Centre for Human Molecular Genetics and Pharmacogenomics, Faculty of Medicine, University of Maribor, Maribor, Slovenia; 3 Department of Anesthesia and Intensive Care, IRCCS San Raffaele Scientific Institute, Milan, Italy; 4 School of Medicine, Vita-Salute San Raffaele Hospital, Milan, Italy; 5 Faculty of Medicine, University of Maribor, Maribor, Slovenia; CHU Nantes, FRANCE

## Abstract

**Introduction:**

The main mechanism of hypotension in septic shock is persistent vasodilation secondary to vascular hyporeactivity despite high endogenous catecholamine levels and despite endogenous activation of the renin-angiotensin-aldosterone system. The classic stepwise approach involves initiation of norepinephrine, up-titration of the dosage to achieve a specified mean arterial pressure and moving to a second-line vasopressor if the patient remains refractory to norepinephrine. This approach often leads to prolonged states of hypoperfusion and high dose catecholamine exposure and is associated with poor clinical outcomes. Given the multifactorial basis of vasodilation in septic shock there is a strong physiological rationale for the early introduction of a multimodal vasopressor strategy that would provide a more physiologically guided approach. This study will compare the effects of a classic stepwise vs. an early balanced multimodal vasopressor strategy in septic shock.

**Methods:**

This is a single blind randomized Phase II study. Patients with septic shock will be randomly assigned to control (classic stepwise vasopressor administration, n = 40) versus interventional (balanced multimodal vasopressor administration, n = 40) groups. The study employs a superiority trial design. Patients in the control group will be started on norepinephrine followed by vasopressin. Additional vasoactive drugs will be added as per the clinical team’s decision. In the interventional group, patients will simultaneously receive norepinephrine, angiotensin II and vasopressin at equipotent starting doses. We hypothesize that balanced multimodal vasopressor administration will result in a significant decrease in renin levels compared to the conventional stepwise strategy. Several secondary and exploratory outcome measures will be investigated. Univariate statistical tests with generalized linear modeling will be used to test for significant differences between the groups.

**Discussion:**

The goal of this randomized controlled trial is to test the clinical efficacy of an early multimodal vasopressor strategy in septic shock. It aims to provide new insights and contribute to improved management of vasodilatory states.

**Trial registration:**

ClinicalTrials.gov NCT06155812.

## Introduction

According to current guidelines for septic shock management, norepinephrine is the vasopressor of choice in patients that remain hypotensive despite adequate fluid resuscitation. In patients with norepinephrine-refractory shock, a second vasopressor is usually added [1]. Selection and timing of initiation of secondary vasopressor remains unclear and there is a considerable clinical heterogeneity in practice. An anonymous web-based survey that was accessible to members of the European Society of Intensive Care Medicine (ESICM) showed that the decision to add secondary vasopressor varies considerably among intensive care physicians. Some based their decision on a predefined maximum dose of first-line vasopressor or ineffectiveness in dosage increase, others wanted to limit the side effects of the first vasopressor regardless of the required dose and some wanted to utilize a second vasopressor with an independent mechanism of action [2].

The classic stepwise approach involves initiation of norepinephrine, up-titration of dosage to achieve a specified mean arterial pressure (MAP) and moving to a second-line vasopressor if the patient remains refractory to norepinephrine. A second vasopressor is often added only after high levels of norepinephrine have been reached and the patient is already clearly in a catecholamine refractory state, which leads to prolonged states of hypoperfusion, hyper-lactatemia, excessive catecholamine exposure and poor outcome [3,4].

Current Surviving Sepsis Campaign (SSC) guidelines for the management of sepsis and septic shock give a weak recommendation that vasopressin should be used as a second-line agent. Epinephrine is given a weak grade of recommendation as a third-line agent in patients with inadequate MAP levels despite administration of norepinephrine and vasopressin [1].

In septic shock, there is persistent vasodilation secondary to vascular hyporeactivity despite high endogenous catecholamine levels and despite endogenous activation of the renin-angiotensin-aldosterone system. Endotoxemia and release of proinflammatory cytokines cause a systemic down regulation of alpha- and beta-adrenergic receptors. Interpatient adrenergic receptor genotype differences can contribute to relative hyporesponsiveness to exogenous norepinephrine infusion [5,6].

Vasopressin levels are inappropriately low in patients with septic shock compared to patients with cardiogenic shock and a similar degree of hypotension. In addition to low levels of circulating vasopressin, there is a hypersensitivity to exogenous vasopressin infusion when comparing patients with septic shock to normotensive healthy subjects. These data suggest that there is an absolute and relative vasopressin deficiency in septic shock [7,8]. The threshold for adding vasopressin varies among studies and remains unclear, but it seems reasonable to add vasopressin when norepinephrine base is in the range of 0.25–0.5 μg/kg/min [1].

Currently, there is no bedside test or an established biomarker for the prediction of a hemodynamic response to vasopressin. Measuring vasopressin plasma concentrations is challenging because of the short half-life and ex-vivo instability. The ideal vasopressin concentration for septic shock is not known since there are no differences in plasma vasopressin levels between hemodynamic responders and non-responders [9]. Co-peptin the C-terminal to vasopressin exhibits a strong correlation with plasma vasopressin levels and is easier to assay, but its role as a biomarker of vasopressin responsiveness has not been established yet [10]. The TT genotype of leucyl and cystinyl aminopeptidase is associated with increased vasopressin clearance and was associated with increased 28-day mortality in patients with septic shock [9]. Genotype analysis with next generation sequencing can last anywhere from few hours to days, and since it is critically important to achieve adequate hemodynamic stabilization early, the use of genotype analysis to predict vasopressin responsiveness is not feasible at the moment.

Evidence from both experimental models and human studies indicates that alterations in the renin–angiotensin–aldosterone system during septic shock can occur at three distinct levels. Impaired generation of angiotensin II (Ang II), possibly attributable to defects in angiotensin-converting enzyme activity, enhanced degradation of Ang II by peptidases and/or unavailability of Ang II type 1 receptor due to internalization or reduced synthesis. These alterations can occur either independently or in combination [11–13]. Theoretically, exogenous infusion of Ang II could at least partially counteract these alterations.

ATHOS-3 study has demonstrated that exogenous Ang II effectively increases blood pressure in patients with vasodilatory shock who required more than 0.2 μg/kg/min of norepinephrine to maintain their blood pressure [14].

Regarding identification of patients with Ang II deficiency, a subgroup post hoc analysis of ATHOS-3 data revealed a reduction in 28-day mortality in patients with renin concentrations above the median [15]. However, given a strong association between renin levels and ICU mortality in patients with septic shock and that Ang II may be especially effective in patients with severe vasodilatory shock (APACHE II scores > 30) [14] it is unclear whether renin levels independently predict Ang II responsiveness or if the relationships becomes more confounded with illness severity. A recent study suggested that higher renin levels were associated with decreased Ang II responsiveness and that higher levels of renin despite Ang II infusion were associated with increased mortality [16]. Currently, there is not enough evidence to support the use of renin levels as a marker of Ang II responsiveness.

Serum lactate levels have been commonly used as a marker of tissue hypoperfusion, indicator of treatment response and an indicator of disease severity and outcome. Resuscitation strategies are usually orientated towards reduction and normalization of serum lactate levels [17].

There is an increasing amount of data indicating that renin is a better marker of tissue hypoperfusion and predictor of intensive care unit (ICU) mortality in patients with sepsis and septic shock, even outperforming lactate [18,19]. Renin increased between the first and third day in non-survivors but decreased in survivors [20,21]. The rate of change in renin concentration but not lactate concentration in ICU patients over the first 72 hours was associated with in-hospital mortality [22]. One of the potential benefits of using renin as a marker of tissue hypoperfusion is that renin measurement is not significantly affected by diurnal variation, continuous renal replacement therapy or medications. Surprisingly, beta-blockers or ACE inhibitors do not interfere with measured renin levels [23]. Elevated renin levels could also be used to identify high-risk patients for acute kidney injury (AKI) and the need for renal replacement therapy [24,25]. High renin levels could play an important role in immune system dysregulation in sepsis and septic shock. Prorenin receptor activation on leukocytes stimulates the production of proinflammatory cytokines [26]. In preclinical models, blockade of prorenin receptor improved survival and was associated with lower levels of proinflammatory cytokines [27]. Hypothetically, exogenous Ang II could also serve as an immune system modulator that offsets the proinflammatory effects of high renin levels.

Given the multifactorial basis of vasodilation in septic shock there is a strong rationale for an early introduction of a multimodal vasopressor strategy to counteract the pathophysiological basis of vasodilation in septic shock [28,29].

We designed a study to compare the effects of different strategies (classic stepwise vs. early balanced multimodal vasopressor administration) on tissue perfusion parameters (renin and lactate kinetics) and clinically relevant outcomes. We hypothesize that balanced multimodal vasopressor administration will result in a significant decrease in renin levels compared to the conventional stepwise strategy and will be associated with improvement of clinically relevant outcomes.

## Methods and analysis

### Trial design

**Fig 1:**
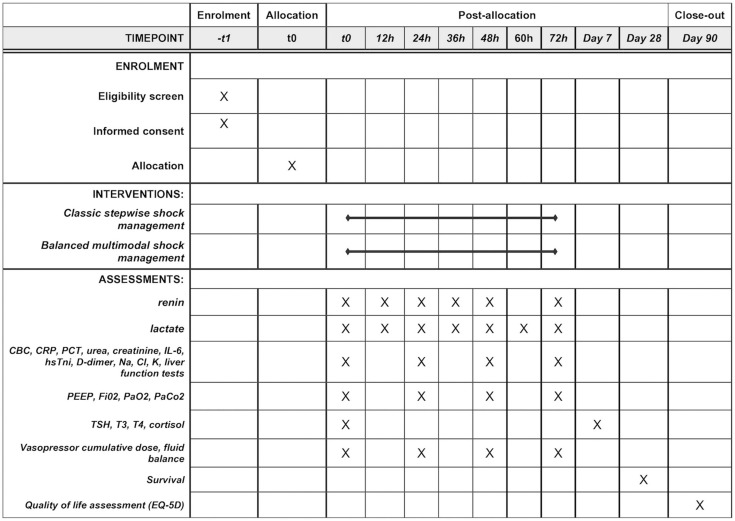
The schedule of enrollment, interventions and assessments.

This study is a multi-centre randomized clinical trial that will compare the effects of classic stepwise vs. early balanced multimodal vasopressor strategy in septic shock. All patients with septic shock will be screened for study eligibility (Fig 1). Participants will be randomly assigned to either the control group (classic stepwise approach) or the interventional group (early balanced multimodal approach). The study is single blinded (patients will be blinded to their allocated group).

### Study setting

The study will take place at three ICUs at University Medical Centre Maribor and University Medical Centre Zagreb. The Medical Intensive Care Unit at the University Medical Centre Maribor will serve as the Study Coordinating Center.

### Inclusion and exclusion criteria

Each patient must meet all the following inclusion criteria to be enrolled in the study:

1.   Adult patients (≥ 18 years).
2. Sepsis (an acute change in total Sequential Organ Failure Assessment (SOFA) score ≥ 2 points consequent to infection) with persisting hypotension requiring vasopressors to maintain MAP ≥ 65 mm Hg and having a serum lactate level > 2 mmol/L despite adequate volume resuscitation (20–30 ml/kg in 3 hours).
3.   Vasopressor requirement ≥ 0.15 μg/kg/min of norepinephrine base equivalent.
4. Patients are required to have central venous access and an arterial line present, and these are expected to remain present for at least the initial 72 hours of study.
5. Patients are required to have a urinary catheter present, and it is expected to remain present for at least the initial 72 hours of study.
6. Patients must have a cardiac index (CI) > 2.3 L/min/m^2^ (measured by bedside echocardiography, pulse contour cardiac output (PiCCO) or Swan-Ganz catheter).

  Patients meeting any of the following exclusion criteria will not be enrolled in the study:

1.   Death expected < 24 hours.
2.   Pregnancy (suspected or confirmed).
3.   Surgery expected for source of infection.
4.   Inter-hospital transfer expected during the first 72 hours of hospitalization.
5.   Liver failure with a Model for End-Stage Liver Disease (MELD) score of ≥ 30.
6.   Patients with acute mesenteric ischemia or a history of mesenteric ischemia.
7.   Patients with Raynaud’s phenomenon, systemic sclerosis or vasospastic disease.
8. Patients with active bleeding and an anticipated need (within 48 hours of initiation of the study) for transfusion of > 4 units of packed red blood cells.
9.   Patients with a known allergy to mannitol (an additional ingredient in Giapreza 2,5 mg/ml).
10. Patients on veno-arterial (VA) ECMO.

### Informed consent

The principal investigator or co-investigators will be responsible for obtaining an informed consent. Emergency consent will be considered. If the patient is unable to provide informed consent because of disease severity, written consent will be obtained from a legal guardian or the closest relative. The informed consent contains basic trial data, details about voluntary nature of participation, the confidentiality of personal data and the right to withdraw at any time. All forms are approved by the ethics committee. If requested, patients will be provided with a copy of the informed consent document.

### Allocation

Sealed envelopes will be used for concealment of the allocation. The envelopes will contain information about the assigned study arm and will be opened after the written consent form is obtained. The allocation sequence will be randomized with the use of True Random Number Generator. The allocation ratio is 1:1. The generation of a randomization sequence will be the responsibility of an independent statistician. Trial participants will be blinded to the allocation group.

### Interventions

Patients who meet all the inclusion criteria and have a vasopressor requirement of ≥ 0.15 μg/kg/min equivalent of norepinephrine base will be eligible for trial enrollment. Patients will be enrolled within 12 hours after admission to the ICU and within 24 hours after the diagnosis of septic shock. Once all of the inclusion criteria are met, patients will be randomized without further delay. The maximum allowed time from achieving the required vasopressor threshold to randomization is 12 hours. To exclude patients with a strong non-vasodilatory component of shock the threshold of cardiac index (CI) > 2.3 L/min/m^2^ was chosen based on the ATHOS-3 trial [14].

### Classic stepwise shock management

Regimen: Norepinephrine base increases of 0.05–0.1 μg/kg/min up to 0.5 μg/kg/min, followed by vasopressin (administered at fixed dose of 0.03 IU/min). Vasopressin will be administered when norepinephrine base is in the range of 0.25–0.5 μg/kg/min as per the clinical team’s decision. If MAP remains < 65 mmHg, norepinephrine base will be uptitrated above the dose of 0.5 μg/kg/min until MAP ≥ 65 mmHg. Maximum norepinephrine base dose will be as per the clinical team’s decision. Initiation of additional vasoactive drugs (epinephrine, Ang II, methylene blue, or dopamine) or inotropes (dobutamine, levosimendan or milrinone) will be as per the clinical team’s decision. In case of persistent hypotension, the addition of Ang II will be considered if norepinephrine base dose exceeds 0.5 μg/kg/min and vasopressin is already administered at the dose of 0.03 IU/min. Rescue therapy for hypotensive episodes will be norepinephrine base as per the clinical team’s guidance.

If MAP is ≥ 75 mmHg norepinephrine base will be tapered in decrements of 0.05 μg/kg/min to maintain MAP ≥ 65 mmHg. If the patient is receiving combined norepinephrine base and vasopressin and the dose of norepinephrine base exceeds 0.5 μg/kg/min, norepinephrine base will be decreased in decrements of 0.05 μg/kg/min to maintain MAP ≥ 65 mmHg. Once the dose of norepinephrine base is lower than 0.5 μg/kg/min the discontinuation of vasopressin will be as per the clinical team’s decision. Before discontinuation, vasopressin will first be tapered to 0.015 IU/min. Maintenance of MAP ≥ 65 mmHg until 72 h after randomization, as per the clinical team’s guidance thereafter.

Rationale: We decided on the maximum applied dose of vasopressin based on retrospective cohort study that showed no difference in early hemodynamic responses between doses of 0.03 IU/min vs 0.04 IU/min while higher doses have been associated with increased rates of mortality, cardiac arrest and adverse effects [30]. Since there are no established guidelines for vasopressor tapering and the order of discontinuation, the timing of vasopressin discontinuation is largely at the discretion of the clinical team. We believe that this approach best reflects clinical heterogeneity in current clinical practice.

### Balanced multimodal shock management

Regimen (S1 Fig): Early, simultaneous start of equipotent doses of norepinephrine base, Ang II and vasopressin at a cumulative dose equivalent to 0.15 μg/kg/min of norepinephrine base. Increments of 0.05 μg/kg/min of equipotent doses of all three vasopressors every 3–5 minutes until MAP ≥ 65 mmHg is reached. Vasopressin will be administered at a starting dose of 0.015 IU/min and a maximum dose of 0.03 IU/min. Ang II will be administered at a starting dose of 20 ng/kg/min and a maximum dose of 100 ng/kg/min. The maximum dose of Ang II will be 80 ng/kg/min during the first 3 hours of treatment.

If MAP is ≥ 75 mmHg decrements of 0.05 μg/kg/min of equivalent doses of all three vasopressors until the dose of Ang II is reduced to the starting dose of 20 ng/kg/min. If MAP ≥ 75 mmHg despite infusion of starting doses of all three vasopressors, vasopressin and norepinephrine base will be weaned off. After weaning off norepinephrine base and vasopressin, Ang II will be used in a maintenance dose of 2.5–20 ng/kg/min until weaned off. If the patient requires vasopressor support of ≥ 0.15 μg/kg/min equivalent of norepinephrine base after 72 h we will continue with infusion of norepinephrine base, Ang II and vasopressin. If the patient requires vasopressor support of ≤ 0.15 μg/kg/min equivalent of norepinephrine base after 72 h, vasopressin and/or Ang II will be weaned off and the patient will receive only norepinephrine base.

If MAP < 65 mmHg despite predefined maximum dose of all three vasopressors, norepinephrine base will be uptitrated to achieve MAP ≥ 65 mmHg. Maximum norepinephrine base dose will be as per the clinical team’s decision. Initiation of additional vasoactive drugs (epinephrine, methylene blue, or dopamine) or inotropes (dobutamine, levosimendan or milrinone) will be as per the clinical team’s decision. Rescue therapy for hypotensive episodes will be norepinephrine base as per the clinical team’s guidance. Maintenance of MAP ≥ 65 mmHg until 72 h after randomization, as per the clinical team’s guidance thereafter.

Rationale: There are no established recommendations regarding the initiation threshold for multiple vasopressors. The threshold of 0.15 μg/kg/min equivalent of norepinephrine base represents a low threshold for initiation of multiple vasopressors and was also chosen from a pragmatical standpoint since it allows for easier titration of multiple vasopressors based on equipotent doses of Ang II and vasopressin relative to 0.05 μg/kg/min of norepinephrine base. Equipotent doses will be calculated from the norepinephrine equivalent score proposed by Kotani et al. [31].

The starting dose of 20 ng/kg/min of Ang II is based on the dosage regimen used in the ATHOS-3 study [14]. We allow for the use of a maximum Ang II dose of 100 ng/kg/min after 3 hours which exceeds the European Medicines Agency recommended maximum dose of 80 ng/kg/min and the recommended maintenance dose of 40 ng/kg/min. If doses of Ang II that exceed 40 ng/kg/min are required after 3 hours for the maintenance of MAP, the patient’s overall vasopressor demand exceeds 0.45 μg/kg/min of norepinephrine base equivalence according to our dosage regimen (S1 Fig). Ang II dose of 100 ng/kg/min is equivalent to a relatively low dose of norepinephrine base (0.25 μg/kg/min) [31] and the use of much higher doses for longer time periods was reported in literature [32,33]. Since our objective is to explore the effects of a balanced vasopressor approach, we consider our strategy to be sensible and appropriate from a clinical perspective.

The weaning strategy was devised so that the patient receives a balanced combination of all three vasopressors for the maintenance of MAP ≥ 65 mmHg, even if there is a strong initial hemodynamic response after the initial addition of vasopressin and Ang II. We plan to favor Ang II in a maintenance dose because if there is a strong initial hemodynamic response (as reported with Ang II) [34–36] and the MAP remains ≥ 65 mmHg after down titration and discontinuation of norepinephrine base and vasopressin, the patient is clearly a hyperresponder to Ang II and only low doses of Ang II will be needed for the maintenance of MAP. The maintenance doses of 2.5–20 ng/kg/min are equivalent to less than 0.05 μg/kg/min of norepinephrine base which reduces the overall vasopressor burden.

### Relevant concomitant care permitted or prohibited during the trial

After initial volume status assessment patients with sepsis induced hypoperfusion will be given 20–30 mL/kg of crystalloid fluids within the first 3 hours of presentation. If there is evidence of fluid overload (patients with congestive heart failure, oliguric or anuric dialysis dependent patients, excessive initial fluid administration etc.) or if further administration of intravenous fluids could lead to a respiratory compromise (patients with acute respiratory distress syndrome (ARDS), aortic or mitral valve pathology etc.) a more restrictive fluid resuscitation strategy will be utilized. We will not routinely check for fluid responsiveness or cardiac dysfunction before adding a second vasopressor since these parameters had already been assessed upon study inclusion. Nonetheless fluid status will be assessed daily with integration of clinical parameters (pulmonary rales, lower extremity edema and jugular venous distention) and ultrasound parameters (vena cava diameter respiratory variation, right ventricle diameter, detection of B-lines, stroke volume variation). Invasive hemodynamic monitoring with PiCCO or Swan-Ganz catheter will be used in case of advanced cardiovascular or pulmonary pathology. Once included in the study, if there is deterioration of cardiac function and it is suspected that cardiac dysfunction is the leading culprit for hemodynamic deterioration, inotropes will be considered. Patients will be given balanced crystalloid solution and/or human albumin preparations. Starches or gelatine will not be used. Patients with severe metabolic acidosis (pH ≤ 7.2) will be given intravenous bicarbonate to maintain pH in the range of 7.2–7.25 until lactic acidosis improves or until dialysis is initiated. We will use a hemoglobin transfusion trigger of 70g/L and 90g/L in patients with evidence of chronic coronary syndrome. If the norepinephrine base requirement for maintaining MAP ≥ 65 mmHg will exceed a dose of 0.2–0.3 μg/kg/min patients will be given hydrocortisone of 200 mg per day in divided doses until vasopressor is weaned of and unless there is a stronger indication for the use of methylprednisolone or dexamethasone (cryptogenic organizing pneumonia, chronic obstructive pulmonary disease (COPD) and asthma exacerbation, lung disease in rheumatic disorders etc.). In patients with high levels of interleukin-6 dialysis with *CytoSorb*® will be considered. We will not routinely use intravenous vitamin C. Lung-protective ventilation strategies (tidal volume of 4–6 ml/kg, plateau pressure ≤ 30 cm H2O and driving pressure ≤ 15 cm H2O) will be applied in patients with acute respiratory failure (patients with pneumonia or non-pulmonary infections resulting in ARDS) who require mechanical ventilation. PEEP levels will be adjusted individually using different assessment tools (P/V tools, stepwise lung recruitment maneuvers, measurement of transpulmonary pressure). If acceptable respiratory and gas exchange parameters cannot be maintained despite the addition of continuous neuromuscular blockade and pronation, veno-venous (VV)-ECMO will be considered. Once VV-ECMO is initiated we will use ultra-protective ventilation strategies. Patients with profound hyperthermia ≥ 39 °C will be managed with a combination of antipyretic medication and physical cooling devices.

### Protocol adherence

Titration of vasopressors to achieve a predefined MAP is the standard of care in critical care units. Every patient will receive a copy of the vasopressor titration sequence for a given MAP according to their allocation group (classic stepwise vasopressor administration or balanced multimodal vasopressor administration). The vasopressor titration sequence will be visible only to authorized personnel in charge of providing medical care. Adherence to the titration sequence will be regularly supervised by the principal investigator and co-investigators.

### Outcome

**Primary outcome measures:**

1.  Change in plasma renin concentration within 72 h after inclusion (continuous variable).

**Secondary outcome measures:**

1.  Change in lactate within 72 hours after randomization (continuous variable).
2. Daily vasopressor dose requirement (maximum daily dose reported as norepinephrine equivalence and duration of vasopressor support (continuous, count variable).
3. AKI rate as defined by the Kidney Disease: Improving Global Outcome (KDIGO) guidelines (continuous variable).
4.  Δ SOFA score between day 1 and day 3 (SOFA score at day 3 minus the baseline SOFA score), (continuous variable).

**Exploratory outcome measures:**

1.  Survival to ICU discharge (binominal variable).
2.  28-day mortality (binominal variable).
3.  Renal replacement therapy requirement during ICU stay (binominal variable).
4.  Quality of life assessment 90 days after ICU admission using EQ-5D standardized questionnaire (ordinal variable).

## Statistical methods

### Sample size

A total of 80 patients will be enrolled. This study employs a superiority trial design to evaluate the hypothesis that the multimodal vasopressor strategy confers superior clinical outcomes compared to a conventional stepwise strategy. The number of enrolled patients was calculated *a priori* in order to ensure the sample size provides at least 80% of statistical power. Power analysis was carried out using GPower 3.1 software [37] with alpha set at 0.05 and using the Wilcoxon-Mann-Whitney test. Effect size (d) was estimated based on the values of renin measurements from studies conducted by Lesnik et al. [20] and Bellomo et al. [15] and was determined as 0.65 and 0.75, respectively. The mean and variances were estimated from median and range values as described elsewhere [38]. To obtain at least 80% of statistical power the sample sizes were estimated as N = 40 for d = 0.65 and N = 30 for d = 0.75, with an expected patient group ratio of ~1.

Given the limited availability of relevant data in this specific context, our calculation focused on observed differences in renin levels rather than a prespecified minimum clinically important difference. We acknowledge this limitation and emphasize that the approach was driven by the data available from the early-phase investigation of this therapeutic strategy.

### Analyzing primary, secondary and exploratory outcomes

The obtained data will be analyzed using IBM SPSS Statistics 25.0 (IBM Inc., Armonk, New York, USA) and R 4.2.3 statistical environment (R Core Team 2020, Vienna, Austria). We will use standard statistical tests to test for significant differences between the control and intervention group in the primary, secondary end exploratory outcomes. An intention-to-treat (ITT) principle will be used. We will summarize data using descriptive statistics for continuous, categorical and ordinal variables. Subgroup analysis with intended subgroups of AKI-RRT (renal replacement therapy) at study inclusion time and prior RAAS inhibitor and/or beta blocker exposure are planned. The Kolmogorov-Smirnov test will be used to assess the normality of data distribution. The χ2 test or Fisher’s exact test will be used to analyze categorical variables. Student’s t-test, one-way ANOVA, Mann–Whitney U-test or Kruskal Wallis H-test will be used to analyze continuous variables between groups. Associations between continuous variables will be determined using Pearson or Spearman’s correlation tests. In case of adjustments or corrections of statistical calculations, generalized linear models will be fitted. Outcome measurements such as renin levels are collected at multiple time points; thus, single time points and trends will be used for the primary analysis. Therefore, the outcome variable in the GLM (generalized linear model) reflects one observation per participant, and no within-subject correlation needs to be accounted for in that analysis. For any analyses involving repeated measurements over time, we plan to employ repeated measures analyses and mixed-effects linear models (MLM) to account for within-subject correlation. Additionally, a blocking technique will be used where appropriate to control for and adjust for subject-level variability and ensure more accurate estimation of treatment effects over time. Significance level will be set at P < 0.05. In case of multiple comparisons, Bonferroni or false discovery rate correction will be applied.

### Missing data

For the primary and secondary outcomes, every effort will be made to minimize missing data. If data are missing, they will be treated as a missing variables with subsequent pair wise exclusion. Sensitivity analyses will be performed to assess the robustness of the results in the presence of missing data. These will include complete case vs. available case comparisons and best-case/worst-case scenario analyses to explore the potential range of outcome estimates. This approach will allow us to evaluate the impact of missing data without relying on imputation methods.

### Interim analysis

An interim analysis will be performed when a halfway mark of patients is achieved (n = 40). A independent statistician will perform an analysis focused on adverse effects associated with vasopressor toxicity and survival parameters. If safety concerns are reported a safety analysis will be paired with an interim analysis of the efficacy of the study’s primary and secondary outcome measures for better context. If required, the final decision on whether to prematurely stop the trial will be made by the Data and Safety Monitoring Board with consultation from the National Medical Ethics Committee of the Republic of Slovenia.

### Safety monitoring

Adverse effects indicative of excessive vasoconstriction include mesenteric ischemia, digital ischemia and necrotic skin lesions [39]. Adverse effects reported with vasopressin use include hyponatremia, fluid retention and cardiac ischemia due to reduced coronary blood flow [9]. Norepinephrine use is associated with a higher incidence of tachyarrhythmias [39]. Ang II use was associated with a higher incidence of venous thromboembolisms [40].

We will continuously monitor for adverse effects associated with vasopressor toxicity and event details will be collected.

### Termination of treatment and/or study participation

Patients may be withdrawn from the study for clinically significant concurrent illness (per investigator determination), occurrence of an adverse effect, patient or legal guardian request, major protocol violation or changes in the patient’s condition making further study treatment unacceptable (per investigator determination).

### Data collection and management

#### Primary, secondary and exploratory outcome assessment.

Renin will be measured at baseline and 12, 24, 36, 48 and 72 hours after randomization. Lactate will be measured at baseline and 12, 24, 36, 48, 60 and 72 hours after randomization. Basic demographic data (age, sex), [41] survival data (survival to ICU discharge, 28-day mortality), daily laboratory data (full blood count, C-reactive protein (CRP), procalcitonin (PCT), urea, creatinine, high sensitivity troponin I (hsTnI), Na, K, Cl, lactate, blood gas analysis, liver function tests, coagulation tests, D-dimer), daily fluid balance, daily interleukin-6 (IL-6) for 72 h after randomization. Daily vasopressor dose requirement. Daily hemodynamic data (systolic and diastolic arterial pressure, MAP). Daily ventilator parameters (PEEP; FiO2) and gas exchange parameters (PaO2 and PaCO2) if applied. Thyroid function tests and cortisol levels on day 1 after randomization and day 7 (if still treated in the ICU).

### Data management

The principal investigator and co-investigators will be responsible for data custody. Collected data will be electronically stored in the hospital management system and physically stored in the study center archives. Only authorized medical personnel will have access to study data.

### Record retention

In accordance with Good Clinical Practice (GCP) guidelines, the principal investigator will retain all documents related to the clinical trial, including patient medical records, signed informed consent forms, ethical committee approvals, and other relevant materials, for a period of 5 years following the conclusion of the clinical trial.

### Confidentiality

Research reports are strictly prohibited from identifying subjects by their names. These reports are solely intended for research purposes. Efforts will be exerted to maintain the confidentiality of subjects’ personal medical data.

### Ethics

Before the trial was initiated, this trial received ethical approval from the National Medical Ethics Committee of the Republic of Slovenia (approval number: 0120–212/2023/5). Written, informed consent to participate will be obtained from all participants.

### Discussion

To the best of our knowledge this is the first RCT that aims to compare the classic stepwise vasopressor administration vs. the early triple vasopressor administration strategy in septic shock and the effects on tissue perfusion parameters. In septic shock patients requiring high-dose vasopressors, there is a lack of evidence regarding optimal therapeutic strategies.

A meta-analysis of thirty-two trials comparing epinephrine, phenylephrine and vasopressin or terlipressin to norepinephrine showed no survival benefit [42]. Regarding combination therapy, the VASST trial (comparing norepinephrine alone vs. norepinephrine plus vasopressin) showed no improvement in overall 28-day mortality [43]. A subgroup analysis showed improved survival with the addition of vasopressin in patients with less severe shock (norepinephrine dose < 15 μg/min) however since many statistical tests were performed the statistical significance of these findings remain uncertain [43]. A systematic review of 10 RCTs done by the SSC campaign showed that the combination therapy of vasopressin with norepinephrine reduced mortality as compared with norepinephrine alone, but the threshold for adding vasopressin varied considerably among studies [1]. Recently, a retrospective observational cohort study showed that vasopressin initiation with low-dose norepinephrine (< 0.25 μg/kg/min) was associated with improvement in 28-day mortality [44].

Ang II appeared to be similar in efficacy compared with norepinephrine in terms of ability to maintain perioperative MAP in cardiac surgery patients with similar overall major adverse outcomes [45]. A pilot study comparing Ang II as a primary vasopressor with other conventional vasopressors (norepinephrine, vasopressin, metaraminol, epinephrine, or combinations) showed similar hemodynamic effectiveness and similar rates of thromboembolic complications [46]. An exploratory post-hoc analysis of ATHOS-3 study data suggested a potential benefit of Ang II introduction at lower doses of other vasopressor agents [47].

The aforementioned data indicate that starting Ang II and vasopressin early could reduce patient mortality in septic shock.

Our decision for the choice of renin as the primary outcome is based on studies that have shown that renin is likely a better marker of tissue hypoperfusion and predictor of intensive care unit (ICU) mortality in patients with sepsis and septic shock compared to lactate [20–22]. The repeated timepoints were chosen to improve repeatability and reproducibility of the study. The time point of 72 hours after randomization was chosen based on studies that have shown that renin increases between the first and third day in non-survivors but decreases in survivors and that rate of change in renin concentration over the first 72 hours was associated with in hospital mortality [20–22]. Given the scarcity of available sources with comparable interventions and outcome measures at the design of the study, we decided against narrower or broader timing windows since, from a clinical perspective, the results would prove difficult to interpret.

We are aware that Ang II infusion will influence the blood renin concentration and that this could be seen as a possible limitation of our study. To offset this limitation, we plan to compare the change in renin levels with clinically relevant outcomes. The use of Ang II does not unequivocally lead to renin decrease and high levels of renin despite Ang II use are associated with increased mortality [16]. We believe that it is not the use of Ang II itself, but the early multimodal vasopressor approach that will lead to a faster trend in renin decrease and to clinically favorable outcomes.

Since no RCT clearly established superiority of a specific vasopressor as a primary choice we hypothesize that early administration of vasopressors with distinct mechanisms of action could prove clinically beneficent with earlier adequate hemodynamic stabilization and positive impact on tissue perfusion parameters and clinically relevant outcomes.

We are aware that our intervention strategy is not personalized and therefore does not acknowledge differences in host genotype, variations in endogenous degradation of Ang II or vasopressin, and variations in organ-specific receptor expression or downregulation. Given that the role of biomarker-guided vasopressor initiation has not been unequivocally established [9,10,15,16] and there is currently no bedside test available for predicting hemodynamic response to catecholamines, vasopressin or Ang II, we believe our strategy to be clinically justified. The insight gained from this study could ultimately contribute to the optimization of therapeutic strategies in septic shock.

### Trial status

At the time of submission, the enrolment of patients had already begun in two participating sites. The first subject was enrolled on December 23, 2023. Enrolment is expected to be completed in late 2025. Data collection will conclude 90 days after enrolment of the last patient. Study results are expected within six months after data collection is completed. This protocol is version 1.9 dated August 2025.

## Supporting information

S1 Checklist SPIRIT 2025 checklist. (DOCX)

S1 Fig Vasopressor titration regimen in the balanced multimodal group. NE = norepinephrine, AVP = arginine vasopressin, Ang II = angiotensin II.
(TIF)

S1 Appendix Ethical approval – english version of approved study protocol. (PDF)

S2 Appendix Consent form. (PDF)

## Abbreviations

MAP: Mean arterial pressure
RCT: Randomized controlled trial
SSC: Surviving sepsis campaign
AT II: Angiotensin II
ACE: Angiotensin-converting enzyme
AT: Angiotensin
ICU: Intensive care unit
AKI: Acute kidney injury
SOFA: Sequential organ failure assessment
CI: Cardiac index
PiCCO: Pulse contour cardiac output
MELD: Model for end-stage liver disease
ECMO: Extracorporeal membrane oxygenation
ARDS: Acute respiratory distress syndrome
COPD: Chronic obstructive pulmonary disease
PEEP: Positive end-expiratory pressure
KDIGO: Kidney disease: improving global outcomes
FBC: Full blood count
CRP: C-reactive protein
PCT: Procalcitonin
HsTni: High sensitivity troponin I
IL-6: Interleukin 6
GCP: Good clinical practice
