# Supplementary figures and images for: Early multimodal vasopressor strategy in septic shock (TRICYCLE)—Study protocol for a randomized controlled clinical trial
Source: PLoS One. 2025 Aug 29;20(8):e0331304. doi: 10.1371/journal.pone.0331304 (PMC12396702; doi:10.1371/journal.pone.0331304)

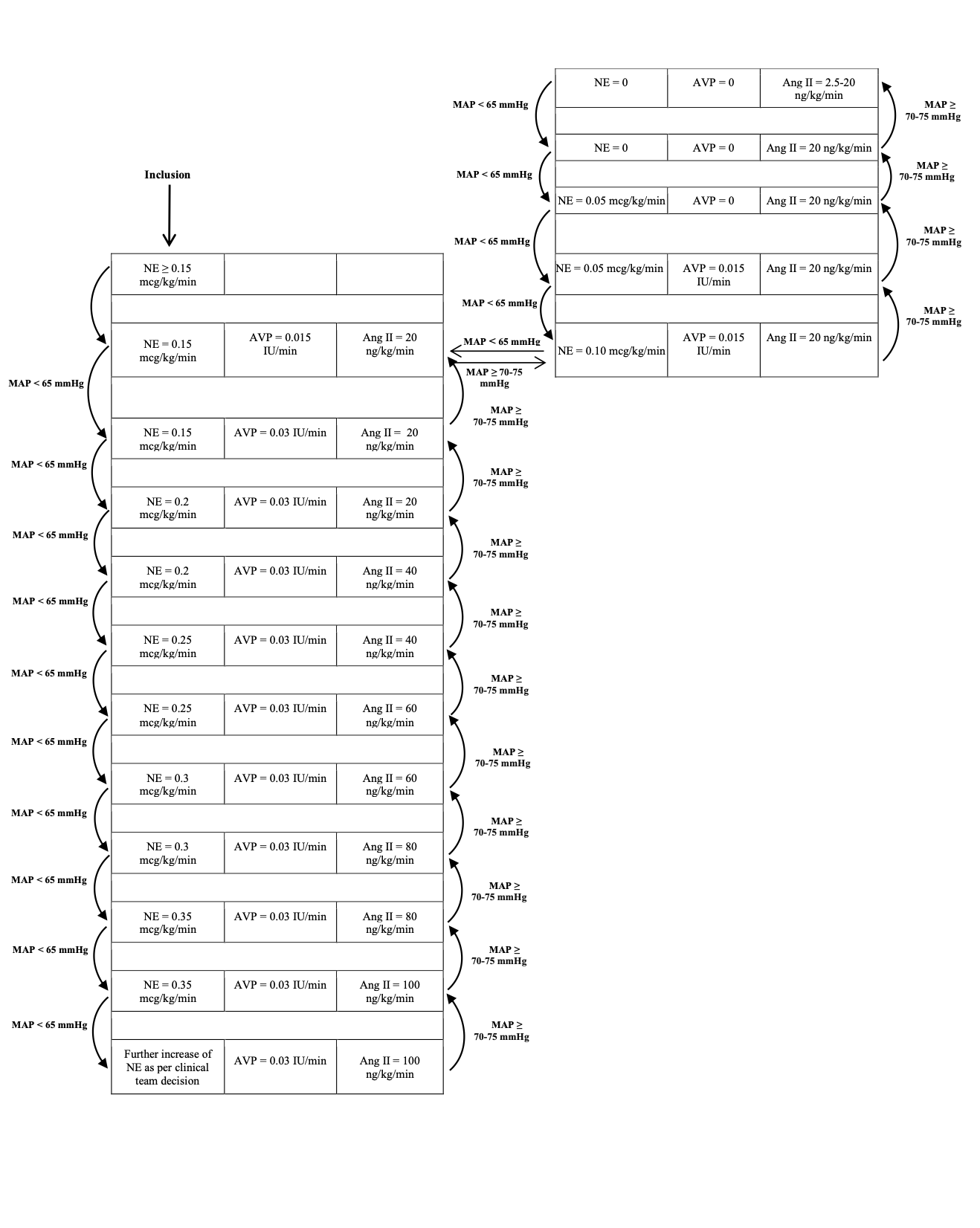

Supplement: S1 Fig — NE = norepinephrine, AVP = arginine vasopressin, Ang II = angiotensin II. (TIF) [file pone.0331304.s002.tif]
