# Supplementary material for: Early multimodal vasopressor strategy in septic shock (TRICYCLE)—Study protocol for a randomized controlled clinical trial
Source: PLoS One. 2025 Aug 29;20(8):e0331304. doi: 10.1371/journal.pone.0331304 (PMC12396702; doi:10.1371/journal.pone.0331304)
Supplement: S1 Appendix — (PDF) [file pone.0331304.s003.pdf]

Ethical approval request form – English version of approved study protocol

»Early multimodal vasopressor strategy in septic shock.«

INTRODUCTION

According to current guidelines for management of sepsis and septic shock norepinephrine is the vasopressor of choice in patients that remain hypotensive despite adequate fluid resusitaction. In patients with shock refractory to norepinephrine a second vasopressor is usually added.[1] Selection and timing of initiation of secondary vasopressor remains unclear and there is a considerable clinical heterogenitiy in practice. Anonymous web based survey that was accesible to members of the European Society of Intensive Care Medicine (ESICM) showed that the decision to add secondary vasopressor varies considerably among intensive care specialists. Some based their decision on a predefined maximum dose of first line vasopressor or ineffectiveness in dosage increase, others wanted to limit the side effects of the first vasopressor regardless of the required dose and some wanted to utilize second vasopressor with independent mechanism of action.[2] The classic stepwise approach involves initiation of norepinephrine, up-titration of dosage to achieve a specified mean arterial pressure (MAP) and moving to second line vasopressor if the patient remains refractory to norepinephrine. A second vasopressor is often added only after toxic levels of norepinephrine have been reached and the patient is already clearly in a catecholamine refractory state.

Current guidelines for the manegement of sepsis and septic shock (SSC) give a weak recomendation that vasopressin should be used as a second line agent and vasopressin was also given a strong grade of recommendation as a second line vasopressor in the aforementioned survey.[1, 2]

In recent years angiotensin II (AT II), a novel vasopressor, has emerged and ATHOS-3 study has demonstrated that ATII effectively increases blood pressure in patients with vasodilatory shock who required more than 0.2 mcg/kg/min of norepinephrine to maintain their blood pressure.[3]

The main cause of hypotension in septic shock is loss of vascular tone which is a consequence of complex multifactorial neurohormonal mechanisms that lead to a disrupted balance between vasoconstrictors and vasodilators. The clinically relevant pathophysiology of vasodilatory states includes downregulation of beta adrenergic receptors, relative and absolute vasopressin deficiency in prolonged shock states and decreased angiotensin-converting enyzme activity which leads to a decreased conversion of ATI to ATII.[4, 5, 6] Chawla et all. and Wierusiewski et all. proposed a shift from classic stepwise vasodilatory shock management towards early balanced multimodal vasopressor strategy which would provide a more physiologically guided approach.[4, 7]

We aim to perform a prospective randomized controlled trial to compare the effects of classic stepwise vs. early balanced multimodal vasopressor strategies in septic shock.

The usual standard of care of patients with septic shock in our ICU (Medical intensive care unit, University medical center Maribor) is in accordance with current SSC guidelines[1] and is further detailed in the Appendix.

INTERVENTIONS

CLASSIC STEPWISE SHOCK MANAGEMENT

Regimen: Norepinephrine increases of 0.05-0.1 mcg/kg/min up to 0.5 mcg/kg/min, followed by vasopressin (administered at fixed dose of 0.03 IE/min). If MAP remains < 65 mmHg, norepinephrine should be uptitrated above dose of 0.5 ug/kg/min until MAP ≥ 65 mmHg. Maximum norepinephrine dose as per clinical team. Initiation of additional vasoactive drugs (epinephrine, methylene blue, angiotensin II, dobutamine or dopamine) as per clinical team.

Rescue therapy for hypotensive episodes: norepinephrine as per clinical team. Maintenance of MAP ≥ 65 mmHg until 72 h after randomization, as per clinical team afterwards.

Rationale: The choice of vasopressin as a second line vasopressor is based on the Current guidlines for management of sepsis and septic shock and the survey of members of the European Society of Intensive Care Medicine (ESICM).[1, 2] The threshold for adding vasopressin varied among studies and remains unclear, but it seems sensible to add vasopressin when norepinephrine is in the range of 0.3-0.5 ug/kg/min, which is also practice in our institution. We decided for the vasopressin fixed dosage of 0.03 IE/min since a recent retrospective cohort study showed no difference in early hemodynamic responses between doses of 0.03 IE/min vs 0.04 IE/min and higher doses in some studies have been associated with increased incidence of mortality, cardiac arrest and adverse effects.[8]

BALANCED MULTIMODAL SHOCK MANAGEMENT

Regimen: Early, simultaneous start of norepinephrine, angiotensin II and vasopressin at equivalent starting doses (equivalent to 0.05 mcg/kg/min of norepinephrine). Increments of 0.05 mcg/kg/min of equivalent doses of all three vasopressors every 3-5 min until MAP ≥ 65 mmHg is reached (vasopressin will be administered at a maximum dose of 0.03 IE/min).

If MAP is ≥ 75 mmHg decrements of 0.05 mcg/kg/min of equivalent doses of all three vasopressors until dose of angiotensin II is reduced to starting dose of 20 ng/kg/min. If MAP ≥ 75 mmHg despite infusion of starting doses of all three vasopressors, vasopressin and norepinephrine will be weaned off. After weaning off norepinephrine and vasopressin, angiotensin II will be used in maintenance dose of 5-20 ng/kg/min until weaned off.

If MAP ≤ 65 mmHg despite predefined maximum dose of all three vasopressors (norepinephrine dose of 0.25 mcg/kg/min, vasopressin dose of 0.03 IE/min and AT II dose of 100 ng/kg/min) norepinephrine should be uptitrated above dose of 0.25 mcg/kg/min. Maximum norepinephrine dose as per clinical team. Initiation of additional vasoactive drugs (epinephrine, methylene blue, dobutamine or dopamine) as per clinical team.

Rescue therapy for hypotensive episodes: norepinephrine as per clinical team. Maintenance of MAP ≥ 65 mmHg until 72 h after randomisation, as per clinical team afterwards.

Rationale:

The main mechanism of hypotension in septic shock is persistent vasodilation secondary to vascular hyporeactivity despite high endogenous catecholamine levels and endogenous activation of the renin-angiotensin-aldosterone system. Endotoxemia and release of proinflammatory cytokines cause a systemic down regulation of alpha and beta adrenergic receptors. Interpatient adrenergic receptor genotype differences also contribute to relative hyporesponsiveness to exogenous norepinephrine infusion.[9, 10]

During septic shock endogenous vasopressin levels are inappropriately low compared to patients with cardiogenic shock and a similar degree of hypotension. In addition to low levels of circulating vasopressin, there is a hypersensitivity to exogenous vasopressin infusion when comparing patients with septic shock to normotensive healthy subjects. These data suggest that there is a absolute and relative vasopressin deficiency in septic shock.[11, 12]

Septic shock causes endothelial injury and reduced ACE activity which leads to reduced AT-II levels. Furthermore, downregulation of the AT receptors occurs during sepsis potentially due to proinflammatory cytokines and increased expression of nitric oxide.[13]

Given the multifactorial basis of vasodilation in septic shock there is a strong physiological rationale for an early introduction of multimodal vasopressor strategy which provides a more physiologically guided approach.

Equivalent doses of angiotensin II and vasopressin relative to norepinephrine will be calculated from norepinephrine equivalent score proposed by Kotani et al.[14]

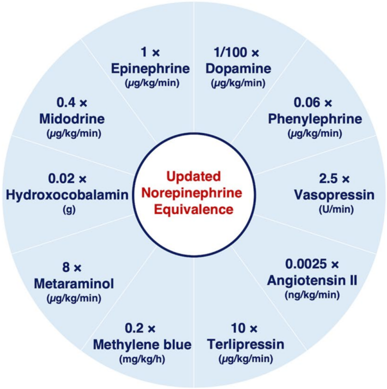

Norepinephrine equivalent score [(14)]

INCLUSION AND EXCLUSION CRITERIA

Each patient must meet all of the following inclusion criteria to be enrolled in the study:

1. Adult patients (≥ 18 years)
2. Sepsis (an acute change in total SOFA score ≥2 points consequent to infection) with persisting hypotension requiring vasopressors to maintain MAP ≥ 65 mm Hg and having a serum lactate level >2 mmol/L despite adequate volume resuscitation (20-30ml/kg in 3 hours).
3. Patients are required to have central venous access and an arterial line present, and these are expected to remain present for at least the initial 72 hours of study.
4. Patients are required to have an urinary catheter present, and it is expected to remain present for at least the initial 72 hours of study.
5. Patients must have Cardiac Index (CI) >2.3 L/min/m^2 (measured by bedside echocardiography, PiCCO catheter or Swan-Ganz catheter).

Patients meeting any of the following exclusion criteria will not be enrolled in the study:

1. Death expected < 24 hours.
2. Pregnancy (suspected or confirmed).
3. Surgery expected for source of infection.
4. Inter-hospital transfer expected during first 72 hours of hospitalization.
5. Liver failure with a Model for End-Stage Liver Disease (MELD) score of ≥ 30 (3 month observed mortality at 52.6 %).
6. Patients on VA-ECMO.

NUMBER OF PATIENTS AND RANDOMIZATION AND DURATION

The aim is to include 80 patients in roughly 2 years. We will use block randomization with variable block sizes. Patients will be randomized preferably within 6 hours after admission to ICU and no later than 24 hours after admission. Futility and safety analysis will be done after 1 year of study or after inclusion of 40 patients. Power calculation is detailed in the paragraph describing statistical analysis.

PRIMARY GOAL

Compare rate of change in renin within 72 h after inclusion.

Rationale: There is an increasing amount of data that renin is the best marker of tissue hypoperfusion and predictor of ICU mortality in patients with sepsis and septic shock, even outperforming lactate. Renin increased between the first and third day in non-survivors, but dropped in survivors.[15, 16] The rate of change in renin concentration but not lactate concentration in ICU patients over first 72 hours is associated with in hospital mortality.[17] One of potential benefits of using renin as a marker of tissue hypoperfusion is that renin measurement is not significantly affected by diurnal variation, continuous renal replacement therapy or medications. Surprisingly beta-blockers or ACE inhibitors do not interfere with measured renin levels.[18] Elevated renin levels could be used to identify high-risk patients for acute kidney injury (AKI) and need for renal replacement therapy.[19, 20]

High renin levels could play an important role in immune system dysregulation in sepsis and septic shock. Prorenin receptor activation on leukocytes stimulates the production of proinflammatory cytokines.[21] In preclinical models blockade of prorenin receptor improves survival and is associated with lower levels of proinflammatory cytokines.[22] Hypothetically exogenous angiotensin II could serve as immune system modulator that offsets the proinflammatory effects of high renin levels.

In the future renin could also serve as a better marker of differentiation between sepsis and septic shock since levels of renin in patients with septic shock are significantly higher compared to patients with sepsis.[15]

SECONDARY GOALS

1. Compare lactate concentration changes within 72 hours after randomization.
2. AKI rate as defined by the Kidney Disease: Improving Global Outcome (KDIGO) guidelines.
3. Compare Δ Sequential Organ Failure Assessment (SOFA) score between day 1, day 3 and day of ICU discharge.

EXPLORATORY ENDPOINTS

1. Survival to ICU discharge.
2. 28-day mortality.
3. Renal replacement therapy requirement during ICU stay.
4. Vasopressor cumulative dose requirement.
5. Quality of life assessment 90 days after ICU admission (using EQ-5D standardised questionnaire).

STUDY DATA

Renin q8h for 72 h after randomization, lactate q8h for 72 h after randomization, basic demographic data (age, sex), survival data (survival to ICU discharge, 28-day mortality), basic daily laboratory data (FBC, CRP, PCT, urea, creatinine, Na, K, Cl, lactate, blood gas analysis), daily fluid balance, daily IL-6 for 72 h after randomization.

PREDEFINED SAFETY DATA

1. Any ventricular or supraventricular tachyarrhythmia requiring any antiarrhythmic therapy.
2. Ischemic events (coronary, mesenteric or cerebrovascular).
3. Deep vein thrombosis (DVT).
4. Pulmonary embolism (PE).
5. Thrombocytopenia.

STATISTICAL ANALYSIS

Number of enrolled patients was calculated *a priori* in order to ensure the sample size providing of at least 80% of statistical power. Power analysis was carried out using GPower 3.1 software[23] with alpha set at 0.05 and using Wilcoxon-Mann-Whitney test. Effect size was estimated based on the values of renin measurements from studies conducted by Bellomo et al.[24] and Lesnik et al.[15] and was determined as 0.65 and 0.75, respectively. The mean and variances were estimated from median and range values as described elsewhere[25]. In order to obtain at least 80% of statistical power the sample sizes were estimated as N=40 and N=30, with expected patient group ratio ~1.

REFERENCES

1. Evans L, Rhodes A, Alhazzani W, et al. Surviving sepsis campaign: international guidelines for management of sepsis and septic shock 2021. *Critical Care Medicine*. 2021;49(11):e1063-e1143.

2. Scheeren TWL, Bakker J, De Backer D, et al. Current use of vasopressors in septic shock. Ann Intensive Care. 2019;9(1):20.
3. Khanna A, English SW, Wang XS, et al. Angiotensin ii for the treatment of vasodilatory shock. N Engl J Med. 2017;377(5):419-430.
4. Wieruszewski PM, Khanna AK. Vasopressor choice and timing in vasodilatory shock. Crit Care. 2022;26(1):76.
5. Russell JA. Vasopressor therapy in critically ill patients with shock. Intensive Care Med. 2019;45(11):1503-1517.
6. Jentzer JC, Vallabhajosyula S, Khanna AK, Chawla LS, Busse LW, Kashani KB. Management of refractory vasodilatory shock. Chest. 2018;154(2):416-426.
7. Chawla LS, Ostermann M, Forni L, Tidmarsh GF. Broad spectrum vasopressors: a new approach to the initial management of septic shock? Crit Care. 2019;23(1):124.
8. Bauer SR, Sacha GL, Lam SW, et al. Hemodynamic response to vasopressin dosage of 0.03 units/min vs. 0.04 units/min in patients with septic shock. J Intensive Care Med. 2022;37(1):92-99.
9. Bucher M, Kees F, Taeger K, Kurtz A. Cytokines down-regulate α1-adrenergic receptor expression during endotoxemia: Critical Care Medicine. 2003;31(2):566-571.
10. Nakada T aki, Russell JA, Boyd JH, et al. B2-adrenergic receptor gene polymorphism is associated with mortality in septic shock. Am J Respir Crit Care Med. 2010;181(2):143-149.
11. Barrett LK, Singer M, Clapp LH. Vasopressin: Mechanisms of action on the vasculature in health and in septic shock: Critical Care Medicine. 2007;35(1):33-40.
12. Demiselle J, Fage N, Radermacher P, Asfar P. Vasopressin and its analogues in shock states: a review. Ann Intensive Care. 2020;10(1):9.
13. Lumlertgul N, Ostermann M. Roles of angiotensin II as vasopressor in vasodilatory shock. Future Cardiology. 2020;16(6):569-583.
14. Kotani Y, Di Gioia A, Landoni G, Belletti A, Khanna AK. An updated "norepinephrine equivalent" score in intensive care as a marker of shock severity. Crit Care. 2023;27(1):29.
15. Leśnik P, Łysenko L, Krzystek-Korpacka M, Woźnica-Niesobska E, Mierzchała-Pasierb M, Janc J. Renin as a marker of tissue perfusion, septic shock and mortality in septic patients: a prospective observational study. IJMS. 2022;23(16):9133.
16. Chung KS, Song JH, Jung WJ, et al. Implications of plasma renin activity and plasma aldosterone concentration in critically ill patients with septic shock. Korean J Crit Care Med. 2017;32(2):142-153.

17. Jeyaraju M, McCurdy MT, Levine AR, et al. Renin kinetics are superior to lactate kinetics for predicting in-hospital mortality in hypotensive critically ill patients*. Critical Care Medicine. 2022;50(1):50-60.
18. Gleeson PJ, Crippa IA, Mongkolpun W, et al. Renin as a marker of tissue-perfusion and prognosis in critically ill patients: Critical Care Medicine. 2019;47(2):152-158.
19. Küllmar M, Saadat-Gilani K, Weiss R, et al. Kinetic changes of plasma renin concentrations predict acute kidney injury in cardiac surgery patients. Am J Respir Crit Care Med. 2021;203(9):1119-1126.
20. Nguyen M, Denimal D, Dargent A, et al. Plasma renin concentration is associated with hemodynamic deficiency and adverse renal outcome in septic shock. Shock. 2019;52(4):e22-e30.
21. Narumi K, Hirose T, Sato E, et al. A functional (Pro)renin receptor is expressed in human lymphocytes and monocytes. American Journal of Physiology-Renal Physiology. 2015;308(5):F487-F499.
22. Hirano Y, Takeuchi H, Suda K, et al. (Pro)Renin receptor blocker improves survival of rats with sepsis. Journal of Surgical Research. 2014;186(1):269-277.
23. Faul F, Erdfelder E, Buchner A, Lang AG. Statistical power analyses using G*Power 3.1: Tests for correlation and regression analyses. Behavior Research Methods. 2009;41(4):1149-1160.
24. Bellomo R, Forni LG, Busse LW, et al. Renin and survival in patients given angiotensin ii for catecholamine-resistant vasodilatory shock. A clinical trial. Am J Respir Crit Care Med. 2020;202(9):1253-1261.
25. Hozo SP, Djulbegovic B, Hozo I. Estimating the mean and variance from the median, range, and the size of a sample. BMC Med Res Methodol. 2005;5(1):13.

APPENDIX

MANAGEMENT OF PATIENTS WITH SEPTIC SHOCK

Septic shock patents are usually admited to our ICU from emergency departement (ED), hospital ward or non tertiary medical centers. After initial volume status assesment patients with sepsis induced hypoperfusion are usually given 20-30 mL/kg of crystalloid fluids within first 3 hours of presentation. If there is evidence of fluid overload (patients with congestive heart failure, oligouric or anuric dialysis dependant patients, excessive inital fluid administration etc.) or if further

administration of intravenous fluids could lead to a sudden respiratory compromise (patients with ARDS, aortic or mitral valve pathology etc.) a more restrictive fluid resusicitation strategy is commonly utilised. Patients are given balanced crystalloid solution or/and human albumin preparations. We do not routinely use starches or gelatine. Patients with severe metabolic acidosis (pH ≤ 7.2) are given intravenous bicarbonate to maintain pH in the range of 7,2-7,25 until lactic acidosis improves or until dialysis is initiated. We commonly use a hemoglobin transfusion trigger of 70g/L and 90g/L in patients with evidence of chronic coronary syndrome.

In patients who remain hypotensive, despite initial fluid resuscitation we use norepinehrine as a first-line vasopressor. Patients that require vasoactive support are routinely administered a central venous access and an arterial line. If the norepinephrine requirement for maintaining MAP ≥ 65 mmHg exceeds dose of 0,2-0,3 mcg/kg/min patients are given hydrocortisone of 200 mg per day in divided doses until vasopressor is weaned of, unless there is a stronger indication for the use of methlyprednisolon or dexamethasone (cryptogenic organizing pneumonia, COPD and asthma exacerbation, lung disease in rheumatic disorders etc.). If the norepinephrine requirement for maintaining MAP ≥ 65 mmHg exceeds dose of 0,3-0,5 mcg/kg/min vasopressin is added. We usually administrate vasopressin at maximum dose of 0.03-0,04 IE/min. If the patients hemodynamic status deteriorates further despite high dose norepinephrine and vasopressin a third line vasopressor (epinephrine or angiotensin II) is added. Use of methylene blue is not a common practice in our ICU . In patients with high levels of interleukin-6 dialysis with *CytoSorb*® is considered. In cases of low cardiac output syndrome dobutamine is considered. We do not routinely use intravenous vitamine C.

We use lung-protective ventilation strategies (tidal volume of 4-6ml/kg, plateau pressure ≤ 30 cm H2O and driving pressure ≤ 15 cm H2O) in patients with acute respiratory failure (patients with pneumonia or non-pulmonary infections resulting in ARDS) that require mechanical ventilation. Intubated patients are positioned in a semi-recumbent position (upper part of bed is elevated 30–45°). We adjust PEEP levels induvidually using different assesment tools (P/V tools, stepwise lung recruitement maneuvers, measurement of transpulmonary pressure).

If we cannot maintain acceptable respiratory and gas exchange parameters despite addition of continuous neuromsucular blockade and proning, VV-ECMO is considered. Once VV-ECMO is initated we use ultra-protectitve ventilation strategies.

For continous analgosedation we predominantly use a combination of fentayl and propofol. If higher doses of propofol (≥ 2,5 mg/kg/h) are required to maintain adequate sedation, midazolam is usually coadministered. Occasionally we decide for volatile sedation with sevoflurane.

In patients presenting with hypoxia without hypercapnia that do not require mechanical ventilation we prefer the use of of high flow nasal oxygen over noninvasive ventilation.

Patients with profound hyperthermia ≥ 39 °C are routinely managed with combination of antipyretic medication and physical cooling devices.
