# Supplementary material for: Early multimodal vasopressor strategy in septic shock (TRICYCLE)—Study protocol for a randomized controlled clinical trial
Source: PLoS One. 2025 Aug 29;20(8):e0331304. doi: 10.1371/journal.pone.0331304 (PMC12396702; doi:10.1371/journal.pone.0331304)
Supplement: S2 Appendix — (PDF) [file pone.0331304.s004.pdf]

PRIVOLITEV ZA SODELOVANJE V MEDICINSKI RAZISKAVI

PODATKOVNI DEL

Podatki o pacientu/nalepka

Pacient je prejel kopijo: **DA** **NE**

Posebne zahteve (na primer drugačen jezik/drugačna komunikacijska metoda):

Odgovorni zdravnik, ki daje pojasnilo: ______________________________

Podatki o raziskavi

Naziv raziskave: *»Sočasna uvedba noradrenalina, vazopresina in angiotenzina II pri bolnikih s septičnim šokom«*

Organizacija, ki izvaja raziskavo, enota, naslov, kontakt: Univerzitetni klinični center Maribor, Oddelek za intenzivno interno medicino, Ljubljanska 5, 2000 Maribor, Slovenija, 02/321 2471

POJASNJEVALNI DEL

Naziv raziskave: Sočasna uvedba noradrenalina, vazopresina in angiotenzina II pri bolnikih s septičnim šokom

Glavni raziskovalec in kontaktne podrobnosti: Žiga Kalamar, dr. med., Oddelek za intenzivno interno medicino, Univerzitetni klinični center Maribor, Ljubljanska 5, 2000 Maribor, ziga.kalamar@ukc-mb.si, 02/321 2471

Namen in potek raziskave:

Pri bolnikih s težko potekajočimi okužbami lahko nastane stanje, ko zaradi posledic okužbe odpove več organov ali organskih sistemov. Organski sistem, ki pogosto odpove, je tudi krvožilni sistem; cirkulatorna odpoved. Cirkulatorna odpoved se največkrat kaže s prenizkim krvnim tlakom, zaradi česar ostali telesni organi (ledvice, jetra, prebavila, možgani…) niso ustrezno prekrvljeni. Poglavitna težava je, da prenizkega krvnega tlaka pri cirkulatorni odpovedi ni mogoče zdraviti z infuzijo tekočin – bolniki na infuzijo tekočin niso odzivni. Umrljivost pri nezdravljeni cirkulatorni odpovedi se približa 100%. Cirkulatorno odpoved zdravimo z vazopresorji – zdravila za višanje krvnega tlaka. Na voljo imamo več zdravil, pri trenutnem načinu uporabe pa zdravila dodajamo zaporedno. Zdravilo prve izbire je noradrenalin, ko dosežemo visoke odmerke noradrenalina večinoma dodamo vazopresin, v primeru neodzivnosti na noradrenalin in vazopresin pa lahko dodamo še eno izmed zdravil tretjega reda (angiotenzin II, adrenalin, fenilefrin, metilensko modrilo ipd.). Težava pri standardnem načinu zdravljenja z vazopresorji je, da pred uvedbo vazopresorja drugega (in tretjega) reda že dosežemo zelo visoke odmerke zdravila prvega (oz. prvega in drugega reda), zaradi česar so pri teh bolnikih zelo pogosti stranski učinki vazopresorjev (pospešen srčni utrip, motnje v prekrvavitevi kože in prebavil, motnje v koncentraciji krvnega sladkorja…).

Namen raziskave je primerjava standardnega načina zdravljenja cirkulatorne odpovedi pri okužbi s takojšnjo uvedbo treh vazopresorjev (noradrenalin, vazopresin in angiotenzin II) v nizkih odmerkih. Noradrenalin, vazopresin in angiotenzin II delujejo na različne sisteme (kateholaminski sistem, vazopresinski sistem in renin-angiotenzin-aldosteronski sistem), z uporabo vseh treh zdravil v nizkih odmerkih pa se morda lahko izognemo stranskim učinkom vsakega posameznega zdravila, ki so pri standardni uporabi zelo pogosti.

Opravili bomo prospektivno, randomizirano raziskavo, kar pomeni, da bodo udeleženci naključno določeni za zdravljenje na standarden način (kontrolna skupina) ali s hkratno uvedbo treh vazopresorjev (intervencijska skupina). Bolniki bodo ves čas zdravljenja na oddelku za intenzivno medicino pod neposrednim nadzorom življenjskih funkcij. Pri vseh bolnikih bo ostalo zdravljenje (uvedba antibiotikov, zdravljenje s tekočinami, prehrana, mehanska ventilacija, nadomestno ledvično zdravljenje...) potekalo nemoteno in v skladu z veljavnimi smernicami za zdravljenje bolnikov s sepso.

Čas in kraj raziskave:

Raziskava bo potekala na Oddelku za intenzivno interno medicino, Univerzitetni klinični center Maribor, predvidoma od oktobra 2023 do predvidoma oktobra 2025.

Vaše sodelovanje v raziskavi je prostovoljno in ga lahko kadarkoli prekličete. Vaša odločitev o prenehanju sodelovanja ne bo vplivala na zdravstveno oskrbo, ki bi jo bili sicer deležni ali na odnos zdravstvenega osebja do vas.

PRIVOLITVENI DEL

Osebna privolitev pacienta:

Spodaj podpisani ____(priimek in ime pacienta tiskano) sem prejel jasne in razumljive informacije o raziskavi**:** Sočasna uvedba noradrenalina, vazopresina in angiotenzina II pri bolnikih s septičnim šokom. Vem, kako bo poskrbljeno za mojo varnost v raziskavi in da lahko kadar koli zaprosim za dodatne informacije in jih tudi dobim. Prav tako mi je bilo pojasnjeno, da lahko privolitev prekličem, ne da bi moral/a preklic utemeljiti in ne da bi prenehanje sodelovanja v raziskavi okrnilo mojo morebitno siceršnjo zdravstveno obravnavo.

S podpisom prostovoljno potrjujem svojo pripravljenost za sodelovanje v raziskavi. Dovoljujem tudi, da se moji demografski in zdravstveni podatki uporabijo v anonimizirani obliki v znanstvene namene. Obrazec podpisujem v navzočnosti raziskovalca/raziskovalke.

Datum in ura: ____

Podpis pacienta: ____

Ime in priimek raziskovalca: ____

Podpis raziskovalca: ____

Privolitev po zakonitem zastopniku ali drugih osebah (če pacient ni sposoben odločanja o sebi):

____(priimek in ime zastopnika pacienta tiskano),

____ (razmerje do bolnika),

____(priimek in ime bolnika tiskano) sem prejel jasne in razumljive informacije o raziskavi**: Sočasna uvedba noradrenalina, vazopresina in angiotenzina II pri bolnikih s septičnim šokom**. Vem, kako bo poskrbljeno za varnost v raziskavi in da lahko kadar koli zaprosim za dodatne informacije in jih tudi dobim. Prav tako mi je bilo pojasnjeno, da lahko privolitev prekličem, ne da bi moral/a preklic utemeljiti in ne da bi prenehanje sodelovanja v raziskavi okrnilo siceršnjo zdravstveno obravnavo.

S podpisom prostovoljno dovoljujem sodelovanje v raziskavi in dovoljujem, da se demografski in zdravstveni podatki uporabijo v anonimizirani obliki v znanstvene namene. Obrazec podpisujem v navzočnosti raziskovalca/raziskovalke.

Datum in ura: ____

Podpis zastopnika: ____

Ime in priimek raziskovalca: ____

Podpis raziskovalca: ____
